# Three-Dimensional Analysis of Cell Division Orientation in Epidermal Basal Layer Using Intravital Two-Photon Microscopy

**DOI:** 10.1371/journal.pone.0163199

**Published:** 2016-09-22

**Authors:** Sari Ipponjima, Terumasa Hibi, Tomomi Nemoto

**Affiliations:** 1 Research Institute for Electronic Science, Hokkaido University, Sapporo, Hokkaido, Japan; 2 Graduate School of Information Science and Technology, Hokkaido University, Sapporo, Hokkaido, Japan; NYU Langone Medical Center, UNITED STATES

## Abstract

Epidermal structures are different among body sites, and proliferative keratinocytes in the epidermis play an important role in the maintenance of the epidermal structures. In recent years, intravital skin imaging has been used in mammalian skin research for the investigation of cell behaviors, but most of these experiments were performed with rodent ears. Here, we established a non-invasive intravital imaging approach for dorsal, ear, hind paw, or tail skin using R26H2BEGFP hairless mice. Using four-dimensional (x, y, z, and time) imaging, we successfully visualized mitotic cell division in epidermal basal cells. A comparison of cell division orientation relative to the basement membrane in each body site revealed that most divisions in dorsal and ear epidermis occurred in parallel, whereas the cell divisions in hind paw and tail epidermis occurred both in parallel and oblique orientations. Based on the quantitative analysis of the four-dimensional images, we showed that the epidermal thickness correlated with the basal cell density and the rate of the oblique divisions.

## Introduction

Division of the basal cells in adult mammalian epidermis is important for maintenance of the epidermal structure, as these cells play a role in replenishing eliminated keratinocytes. Several skin diseases, including cancer, atopic dermatitis, and ichthyosis vulgaris, disrupt the balance between the proliferation and elimination of keratinocytes and result in abnormal skin structures [1–3]. Meanwhile, adult epidermal structures differ depending on the body site. In adult mice, dorsal and ear epidermis are structurally similar and consist of only 3–5 layers [4, 5]. In paw epidermis, both the suprabasal compartment and the cornified layer are relatively thicker than the dorsal and ear epidermis. Tail epidermis is also thick and has a very specific structure composed of two distinct regions, which are called scale and interscale [6]. The epidermal structure in each body site is basically maintained after maturation. Recently, an epidermal homeostasis model was proposed, with the assumption that the basal cell number is constant and suggesting that a proliferative basal cell in ears and tails can generate two proliferative basal cells, two differentiated basal cells, or one of each [7, 8]. However, the mechanism for maintaining epidermal thickness has not yet been clarified and may differ among epidermal tissues with different thicknesses. Comparisons of keratinocyte behaviors in the epidermis at various body regions of a living animal are required to investigate the differences in this mechanism.

When investigating cell division, it is important to determine not only the number but also the orientation of the divisions. Oriented cell divisions are involved in the cell fate determination of various cell types, particularly during developmental periods, such as neuroepithelial cells in the cerebral cortex, retinal cells, epicardial cells, and blood vessel endothelial cells [9–13]. In epidermal basal cells, a division parallel to their basement membrane generates two basal cells. In contrast, a perpendicular division generates a basal cell and a suprabasal cell, which is considered to have lost the ability to proliferate. Regulation of cell division orientation is required for maturation from a monolayer to the stratified epidermal structure in embryonic mice [14–17]. Moreover, in adult mice, the loss of atypical protein kinase C (aPKC), a regulator of cellular polarity, affects the cell division orientations and alters epidermal structures [18]. However, it is poorly understood whether cell division orientation is involved in maintenance of the epidermal structure and whether these mechanisms depend on the body region.

Intravital skin imaging using two-photon microscopy has advanced rapidly in recent years and has been applied to research on the dynamics of stem cells or immune cells in mammalian skin [19–25]. However, most of these investigations using skin were performed with ear skin [22]. In particular, non-invasive intravital imaging of the dorsum has been rarely used because of its sensitivity to vibrational motion caused by the heartbeat and breathing. A new intravital imaging approach that compares the skin among body sites was required to compare the cellular behaviors and structures among the epidermis in various body sites and to clarify the relationship between the behaviors and epidermal thickness.

In this paper, we introduce a new intravital imaging method to compare the skin of the dorsum, ear, hind paw, and tail. Furthermore, we show that there is a correlation between the epidermal thickness and the proportion of “oblique” cell divisions using four-dimensional (x, y, and z over time) quantitative analysis.

## Results

### Establishment of intravital skin imaging in R26H2BEGFP hairless mice

**Fig 1:**
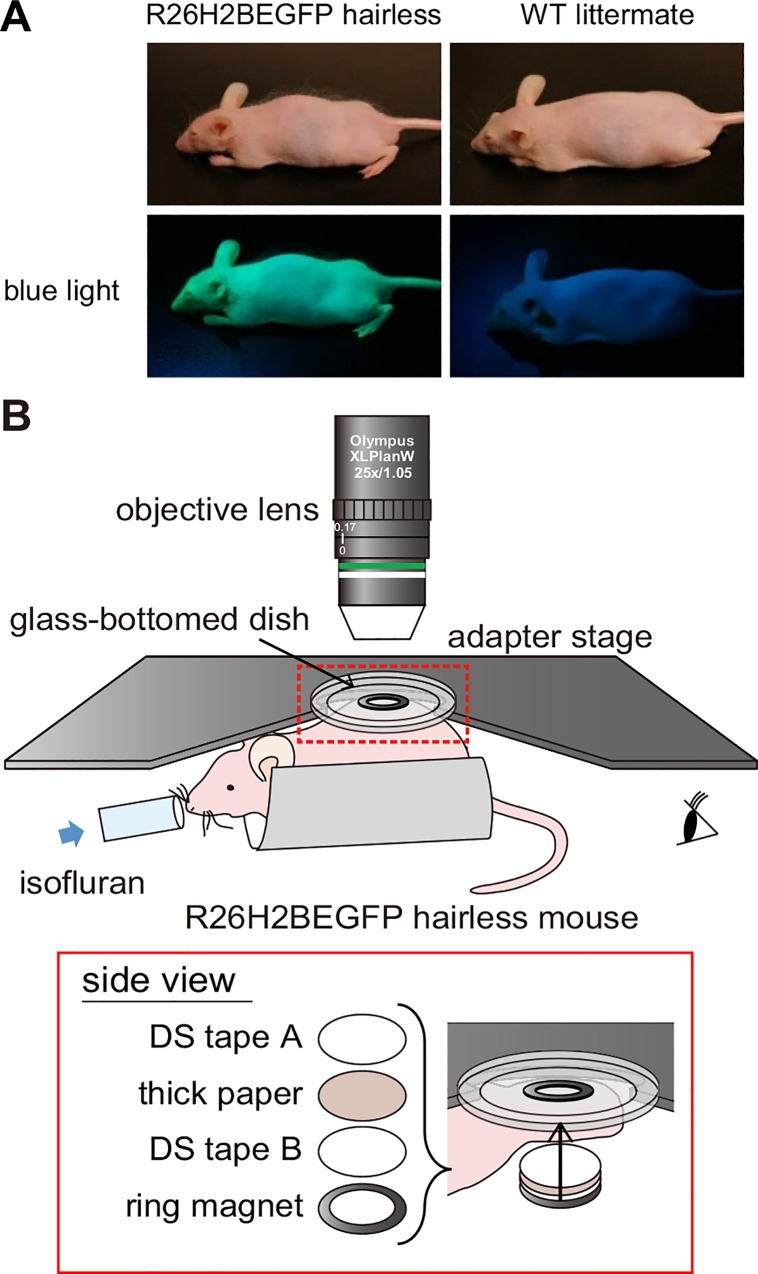
Our intravital imaging method using the newly established R26H2BEGFP hairless mice. (**A**) Photograph of a R26H2BEGFP hairless mouse and a WT littermate. The upper and lower panels show each mouse under ordinary white light and blue light. (**B**) A schematic showing the intravital imaging of the dorsal skin with an upright two-photon microscope. The inset in the red rectangle shows the region indicated by the red dashed rectangle in the upper image viewed from the side. The detailed procedure is provided in the Materials and Methods and S2 Fig.

R26H2BEGFP mice, in which a histone 2B (H2B) and enhanced green fluorescent protein (EGFP) fusion protein is expressed in the nuclei of all cells under the ubiquitous ROSA26 promoter [26], are useful for the visualization of cellular behaviors. However, the hair and melanin in the R26H2BEGFP mice are obstacles to intravital skin imaging [22]. Therefore, we established albino and hairless R26H2BEGFP mice by repeatedly backcrossing these animals with HR-1/Hos mice (hereafter referred to as R26H2BEGFP hairless mice; Fig 1A). In appearance, the mice looked healthy and showed no apparent abnormalities compared with the wild-type (WT) mice (Fig 1A). In addition, the histological and immunostained sections of R26H2BEGFP hairless mice showed that there were no structural abnormalities (S1 Fig). Thus, in this study, we utilized the R26H2BEGFP hairless mice to perform intravital skin imaging of various body regions.

**Fig 2:**
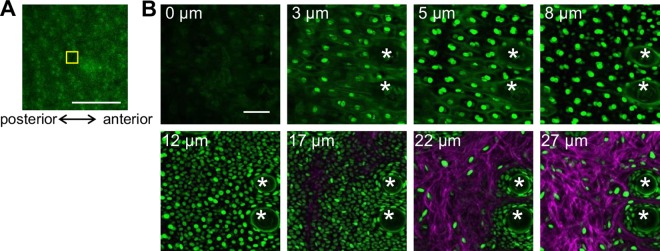
Images of dorsal skin in living R26H2BEGFP hairless mice. (**A**) Low-magnification image of dorsal skin around the field-of-view of the images obtained by confocal microscopy at a 488 nm excitation wavelength. Scale bar = 1 mm. (**B**) A series of optically sectioned images using a two-photon microscope at a 900 nm excitation wavelength at the position indicated by the yellow rectangle in **A**. The depth from the surface of the skin is shown in the upper left of each image. The white asterisks indicate the hair follicles. Scale bar = 50 μm.

**Fig 3:**
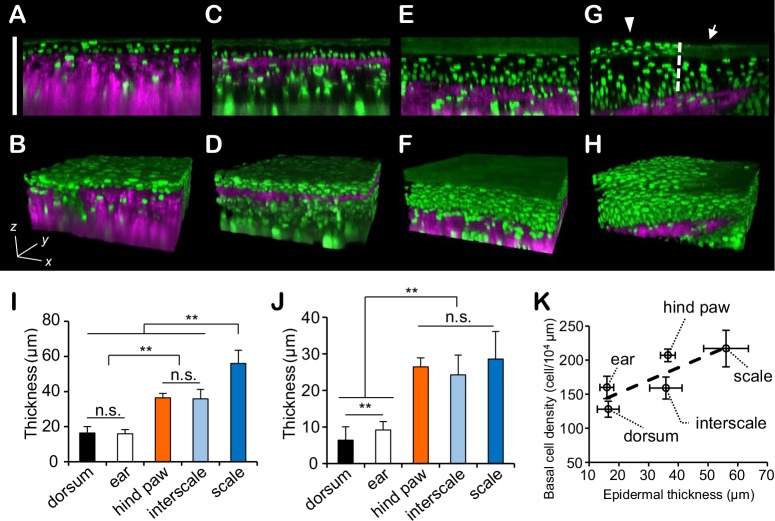
Comparison of the epidermal structures. (**A-H**) Orthogonal images and reconstructed three-dimensional images of the following skin areas: dorsum (**A**, **B**), ear (**C**, **D**), hind paw (**E**, **F**), and tail (**G**, **H**). Scale bar = 100 μm. The arrow and arrowhead show the interscale and scale, respectively. (**I**, **J**) The average epidermal thickness without the cornified layer (**I**) and the thickness of the cornified layer (**J**). These data were obtained from 21 (dorsum), 15 (ear), 15 (hind paw), 14 (interscale), and 14 (scale) points across 5–7 mice per group and compared using the Steel-Dwass test. The error bars represent the standard deviations **P < 0.01; n.s., not significant. See also S3 and S4 Tables. (**K**) The relationship between the basal cell density and epidermal thickness (**I**). The correlation coefficient was 0.837. The dashed line is the regression line. The error bars represent the standard deviations. The statistical significance of the differences in the basal cell densities between the body regions was shown in S5 Table.

We sought to establish a method of stabilizing the observation area of the dorsum without surgical or chemical invasion to compare the epidermis at various body sites in living mice. The dorsal skin of an anesthetized mouse was attached to a glass-bottomed dish and immobilized using a pair of neodymium ring magnets, two types of double-sided (DS) tapes (DS tape A and B), and thick paper (Fig 1B and S2 Fig, and see Materials and Methods) to suppress the influence of movement. In the case where only a pair of ring magnets was used, the skin color inside the ring magnet became paler than the skin outside the ring magnet due to the strong magnetic force. Thus, one or two sheets of thick paper were inserted between the skin and the lower ring magnet to weaken the magnetic force until the skin inside the ring magnet was no longer pale. Using this method of gentle (yet tight) stabilization, we were able to visualize three-dimensional structures in healthy dorsal skin using a two-photon microscope (Figs 2, 3A and 3B). In epidermal cells located at progressively deeper levels, the lateral size of the nucleus became smaller and the cell density gradually increased (Fig 2B). The basal cells, which are located just above the basement membrane, were identified by referring to the second harmonic generation (SHG) signals from the collagen fibers in the dermis. In dermis, there was a small population of cells that were presumed to be fibroblasts, glandular cells, or some type of immune cell. These results indicate that our new approach enabled three-dimensional observations of the keratinocytes in all layers of epidermis and the collagen fibers of the dermis in the dorsum of a living mouse.

The skin of the ear, hind paw, and tail was observed non-invasively using two-photon microscopy to compare the epidermal structures and cellular behaviors among the different body sites. These body sites could be stably attached to a glass-bottomed dish with plastic tape. Even for thick epidermis, such as the hind paw and tail, the structures could be visualized three-dimensionally (Fig 3C–3H and S3 Fig). Interestingly, in tail epidermis, the parakeratotic scale region could be distinguished from the orthokeratotic interscale region by the specific retention of the nuclei in the cornified layer, which is a known characteristic of parakeratosis, using the H2BEGFP signals. For quantitative comparison, the thickness and basal cell density of the five types of epidermis were measured from reconstructed three-dimensional images. The thickness was consistent with the values measured from the histological sections of all body sites (Fig 3I and 3J and S1B and S1C Fig). In addition, the basal cell density correlated with the epidermal thickness, with a correlation coefficient of greater than 0.83 (Fig 3K). This finding suggests that the basal cell density is related to the mechanism that maintains the epidermal thickness.

### Visualization of basal cell division by four-dimensional intravital imaging

**Fig 4:**
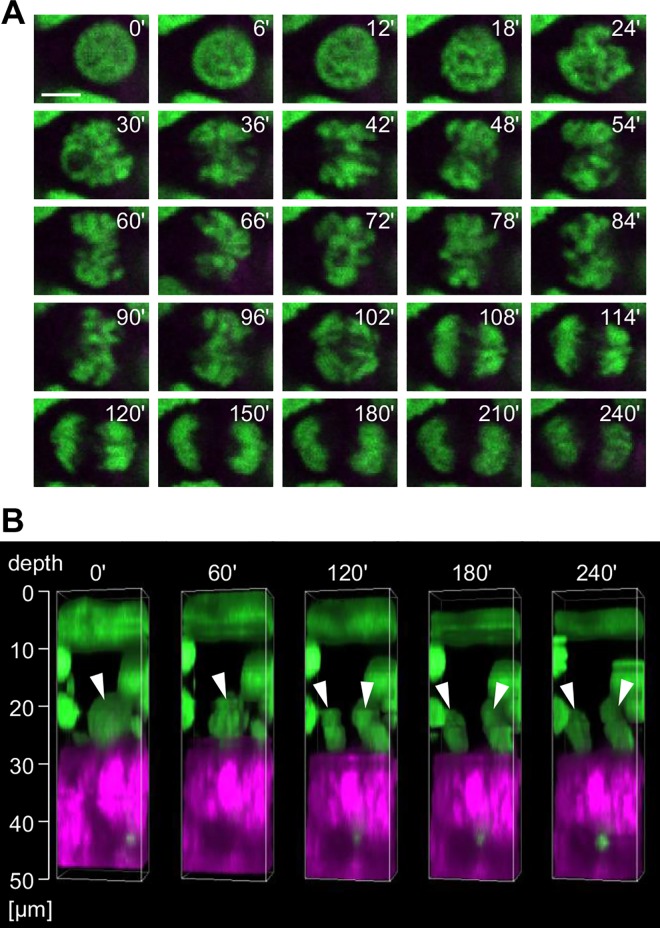
Four-dimensional imaging of cell division in the dorsal epidermis. (**A**) Time-lapse images of the basal cells of the dorsum at a 24-μm depth from the skin surface. Images that were recorded every 6 min or 30 min are shown. These images were detected with sufficient sensitivity to determine the mitotic stage from the pattern of chromatin. It is presumed the cell is in prophase at 0–18 min, prometaphase at 24–36 min, metaphase at 42–96 min, early anaphase at 102 min, anaphase at 108–120 min, and probably telophase at 120–240 min. Scale bar = 5 μm. (**B**) Reconstructed three-dimensional images. The white arrowheads indicate the dividing cell. Images taken every 60 min from 0 min to 240 min in **A** were used for the reconstruction.

The use of four-dimensional imaging over 4 hours in the dorsum and ear enabled the visualization of the morphological changes in chromosomes during mitosis (Fig 4A and S4 Fig, and S1 Movie). In detail, the sequence of events in mitosis was visualized as follows: the chromosomes aligned in metaphase, divided, moved to opposite sides, and stopped in anaphase. Subsequently, in telophase, the two groups of chromosomes formed an internal concave shape (similar to a pair of cashew nuts). Some cells in which a group of chromosomes gradually formed an ellipsoid were subsequently observed. Remarkably, in dorsal and ear epidermis, the two daughter cells seemed to be located at the same depth as the parent cell, namely, they divided approximately parallel to their basement membrane (Fig 4B and S2 Movie). If the number of the basal cells is constant, cell migration from the basal layer to the suprabasal layer should occur simultaneously with parallel division. However, we could not find any basal cells migrating from the basal layer into the suprabasal layer in our observations, although we could find some laterally and rapidly migrating cells in the epidermis and dermis that were presumed to be some type of immune cell (S1 Movie).

**Fig 5:**
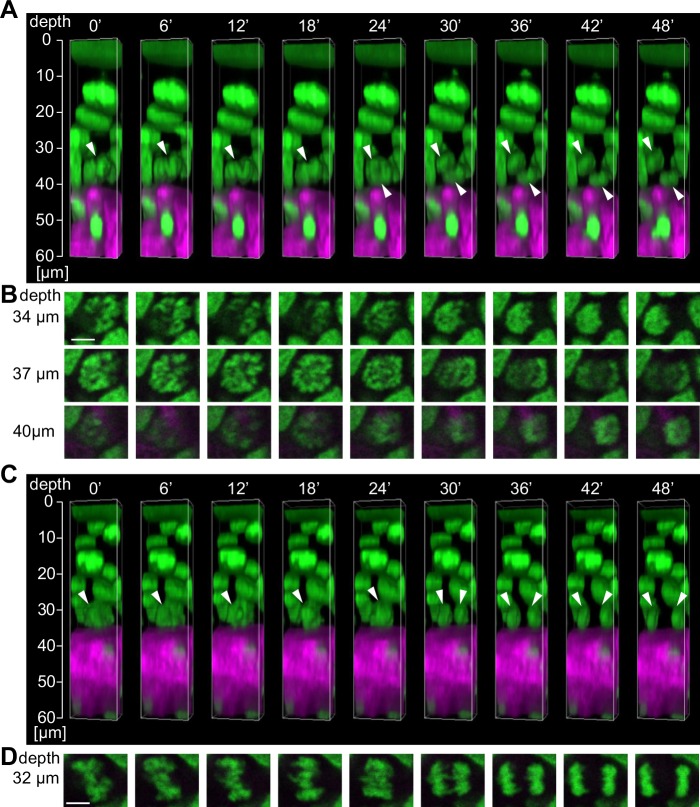
Four-dimensional imaging of cell division in the hind paw epidermis in a living mouse. (**A**) Reconstructed three-dimensional images of the oblique division to the basement membrane from images taken every 6 min. The white arrowheads indicate a dividing cell. (**B**) Time-lapse images of the mitotic cells shown in **A** in the *x*-*y* plane at each depth. Scale bar = 5 μm. (**C**) Reconstructed three-dimensional images of the division parallel to the basement membrane from images taken every 6 min. The white arrowheads indicate the dividing cell. (**D**) Time-lapse images of the mitotic cells shown in **C** in the *x*-*y* plane at a depth of 32 μm from the skin surface. Scale bar = 5 μm.

Four-dimensional imaging was also applied to the hind paw and tail. Mitotic cells were successfully visualized, even in the thick epidermis (Fig 5, S3 and S4 Movies). From the images of both the hind paw and tail, we noticed the presence of both parallel divisions and “oblique” divisions that were not observed in the dorsum and ear. In the case of oblique division, one set of chromosomes was directed upward and the other set was directed downward from the location of the cell prior to division. Based on the position of the two divided nuclei, it was conceived that the upward daughter cell was located in the suprabasal layer, whereas the downward daughter cell stayed in the basal layer. Similar to the dorsum and ear, we could not find any basal cells that were migrating into the upper layer in the hind paw and tail. These results suggest that the upward translocation of the basal cells in the thick epidermis is at least in part mediated by the oblique cell divisions.

### Quantitative analysis of cell division orientation revealed differences according to body site

The three-dimensional orientations of the cell divisions were quantitatively analyzed to investigate the relationship between the epidermal thickness and the cell division orientations. First, the lateral component of the orientation was measured as an angle between the reference line in each epidermis and a line that connects the center of two nuclei of daughter cells during late anaphase or telophase (S5 Fig). The mean angles and the standard deviations in each region were 34.8 ± 26.3° in the dorsum, 54.0 ± 22.0° in the ear, 34.4 ± 25.7° in the hind paw, 57.8 ± 25.9° in the interscale, and 49.4 ± 21.3° in the scale. Although slight biases seemed to exist in the distributions according to body site, the standard deviations were high in all regions. Thus, these results suggested that the lateral components of the cell division orientation in all body sites are broadly distributed, without appreciable bias.

**Fig 6:**
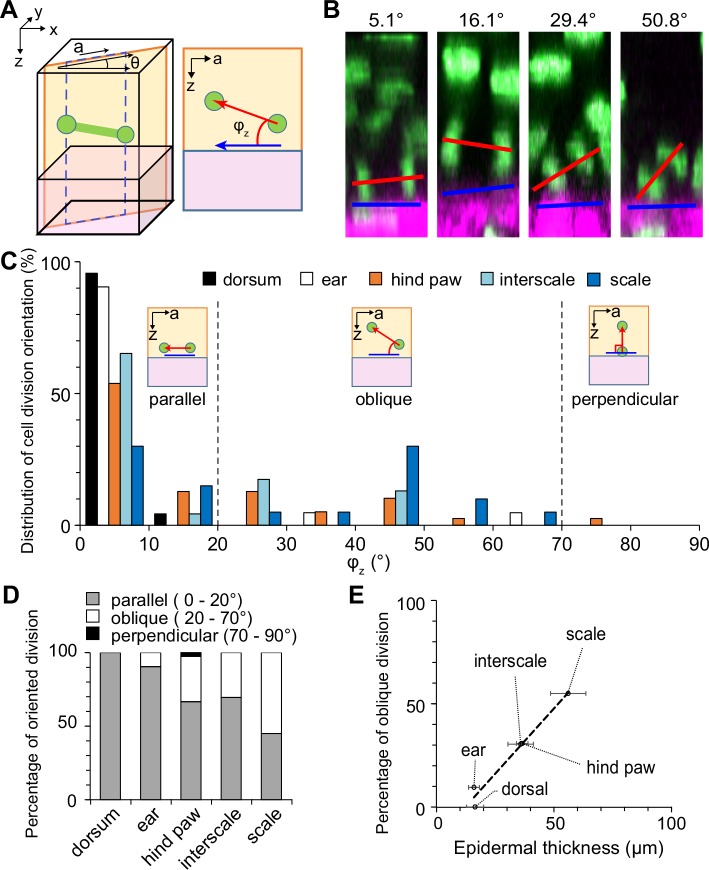
Analysis of cell division orientations relative to basement membrane. (**A**) Schematic of the cell division orientation measurements. The direction “*a*” is defined as the lateral (on the *x*-*y* plane) component of the cell division orientation. The angle was measured in the *a*-*z* plane. (**B**) Examples in the hind paw. (**C**) Histogram of the cell division orientations obtained from 23 (dorsum), 21 (ear), 39 (hind paw), 23 (interscale), and 20 (scale) divided cells. (**D**) The percentage of parallel (*φ*_*z*_ ≤ 20°), oblique (20° < *φ*_*z*_ ≤ 70°) or perpendicular (70° < *φ*_*z*_ ≤ 90°) divisions in **C**. (**E**) Relationship between epidermal thickness (Fig 3I) and the percentage of oblique divisions (**D**). The correlation coefficient was 0.986. The dashed line is the regression line. The error bars represent the standard deviations.

The orientation of cell division relative to its basement membrane was determined as the angle against the border between the epidermis and dermis, as identified by the SHG signals (Fig 6). The mean angles and the standard deviations in each region were 3.9 ± 3.5° in the dorsum, 6.9 ± 13.3° in the ear, 17.0 ± 17.6° in the hind paw, 13.2 ± 14.9° in the interscale, and 28.5 ± 20.9° in the scale. In the dorsal and ear epidermis, >90% of cell divisions were at 0–20°, namely, most cell divisions were approximately parallel to the basement membrane. The similar distributions of the hind paw epidermis and the interscale region indicated that approximately 30% of the cells underwent oblique division (20–70°) and 70% underwent parallel division. The scale region showed a specific distribution pattern, in which more than half of the divisions were oblique. In contrast, perpendicular division (70–90°) was rarely observed in any of the regions. Intriguingly, the percentage of oblique divisions was strongly correlated with the epidermal thickness, with a correlation coefficient of greater than 0.98 (Fig 6E). These results revealed that the distribution of the three-dimensional orientation of cell division depends on the body site and suggested that the oblique divisions of epidermal basal cells relative to their basement membrane are involved in maintaining the epidermal thickness.

## Discussion

In this study, we used two-photon microscopy to perform four-dimensional skin imaging in various body regions of a living mouse, resulting in the visualization of fine morphological changes in chromosomes of the epidermal basal cells in a living mouse. A near-infrared laser light for two-photon excitation can alleviate the influence of scatter, and finer images than the images achieved by confocal microscopy could be obtained, even in the deepest layer of the thick epidermis and the dermis (S6 Fig). Regarding the equipment, highly sensitive detection using the GaAsP non-descanned detectors enabled observations at a high signal-to-noise ratio. Additionally, the curvature of the basement membrane was visualized using SHG signals to examine the three-dimensional orientation of the basal cell division. The use of SHG signals is one of the major distinguishing features of intravital two-photon microscopy.

For two-photon imaging of skin in various body regions, it is essential to use mice that are both albino and hairless. Because melanin absorbs infrared light, many speckles with bright-intensity signals are generated in the image acquired by a two-photon microscope and, in a severe cases, heat injury may be caused [22]. Removal of the hairs with a razor or using a depilatory cream would be needed to observe skin covered with hairs. However, these approaches should be avoided if possible because they might damage the epidermis or cause inflammation at the observation area [22]. Therefore, our established R26H2BEGFP hairless mice are very useful for intravital imaging. Meanwhile, hairless mice have several differences, including hair follicles, compared with commonly used hairy mice [27]. Because previous studies reported that hair follicle stem cells contribute not to homeostasis of the epidermis but to wound repair [28, 29], careful interpretation might be needed, particularly in studies of wound repair. For non-invasive observations, the simple method of stabilization by plastic tape is applicable to the ear, hind paw, and tail, but not to the dorsum, which is sensitive to body motions. If dorsal skin was held firmly with a strong mechanical force, the color of the skin surface would be abnormal and local ischemia would occur. In the ischemic epidermis, the nuclei gradually became distorted and some cells in metaphase stopped dividing (data not shown). It should be noted that the skin might be affected by mechanical stress from the holding instruments, even in methods that do not involve surgical or chemical treatments.

The cell division orientation in the tissue should be analyzed three-dimensionally to clarify its physiological roles in maintaining the epidermis. Using our approach, we could measure the cell division orientations correctly from the direction of movement of two groups of chromosomes during mitosis. In previous studies, the cell division orientations were measured two-dimensionally from tissue sections that were sliced perpendicular to the skin surface. In that case, it was difficult to measure the correct angle because part of the lateral components of the orientation may be lost. This result indicates that some oblique divisions might be mistaken for perpendicular divisions. Of course, three-dimensional analyses of the orientation have been conducted in several previous studies that used fixed whole-mount epidermal tissues from ears and tails [7, 8]. The results of these studies were roughly consistent with our data for the ear or the interscale, but some slight differences exist in the proportions of the orientations and in the interpretation of the data. Notably, a fixation process might have the risk of influencing the cell division orientation or the structures of cells and tissues. Additionally, it is necessary to peel off the epidermis from the dermis before fixation to stain the whole-mounts of the epidermis, which might affect the shapes of the tissues and cells. Regarding the tail data presented in previous reports, the measurement might be performed without distinguishing the orthokeratotic interscale region and the parakeratotic scale region [7]. Because our method could determine the accurate three-dimensional orientations without considering the influence of peeling and fixing, we believe that our intravital three-dimensional measurements are more reliable. Meanwhile, our method cannot rule out the possibility that an upper daughter cell generated by an oblique division contacts the basement membrane, indicating that the upper cell is also a basal cell because only the nuclei are visualized with H2BEGFP. For further clarification, *in vivo* visualization of the plasma membrane could be helpful using techniques such as intradermal injection of a lectin-conjugated fluorescent dye [30] or crossing with another transgenic mouse in which the plasma membrane is labeled [31].

**Fig 7:**
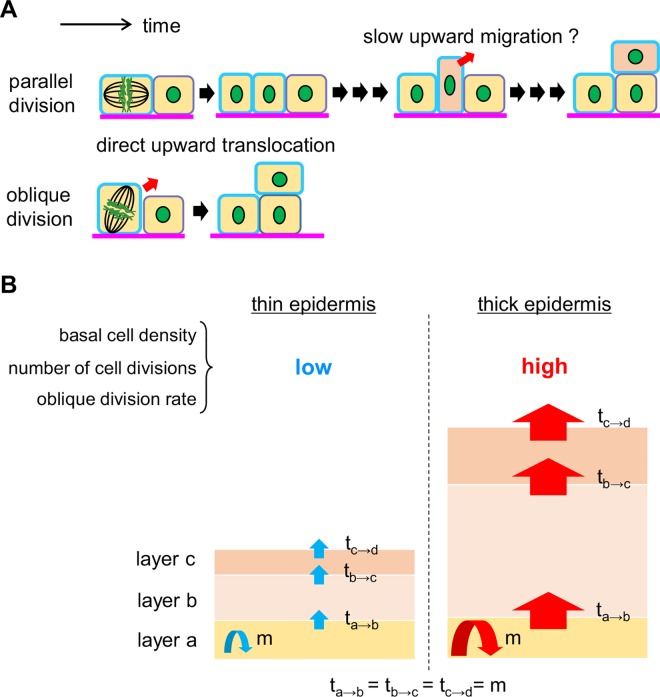
A summarized schematic of the proliferation and migration of the epidermal basal cells, based on our results. (**A**) Schematic of parallel and oblique division. Parallel division generates two basal cells. Because the basal cell density is maintained, the number of parallel divisions is similar to the number of cells migrating to the suprabasal layer. Thus, after a parallel division occurs, a basal cell might gradually migrate into the suprabasal layer over at least 4 hours. On the other hand, oblique division generates one basal cell and another cell that is translocated into a suprabasal layer, without slow migration. Thus, oblique divisions might play an important role in maintaining the rapid upward stream of keratinocytes in thick epidermis. (**B**) A simple model of the maintenance of the thin and thick epidermis. The epidermis can be roughly considered as having three layers: a proliferative basal layer (layer a), a differentiated cell layer (layer b), and a cornified layer (layer c). Assuming that the increase and the decrease in cell numbers in each layer is approximately equal to the number of cell divisions (as described in the text), then the basal cell density, the number of cell divisions, and oblique division rate are increased in thick epidermis compared with thin epidermis. Thus, the upward stream of the keratinocytes in thick epidermis is more rapid than in thin epidermis.

We were able to visualize parallel divisions in the epidermis of all body sites observed in this study. The number of parallel divisions should be similar to the number of cells migrating to the suprabasal layer because the basal cell density in adult mice is known to be maintained [7, 8]. However, we could not identify cells migrating to the suprabasal layer during the 4-hour observation and therefore propose that the cells may undergo extremely slow migration (Fig 7A). In contrast, oblique division does not affect the basal cell numbers, as it generates a daughter cell located in the suprabasal layer. Thus, oblique divisions can supply a cell to the suprabasal layer simultaneously with cell division, without the hindrance of extremely slow migration. Therefore, the role of oblique division in the thick epidermis might be to accelerate the supply of cells to the suprabasal layer. Moreover, our data show a correlation between the basal cell density and the epidermal thickness. In addition, previous studies showed that both the proportion and the number of cycling cells in the basal layer correlate with the epidermal thickness [5, 32]. These data indicate that the number of cell divisions in thick epidermis is much larger than the number of divisions in thin epidermis. As a simple model, the epidermis can be divided into three layers: a proliferative basal layer, a differentiated cell layer, and a cornified layer (Fig 7B). Assuming that the cell number in each layer is constant, all of the increases and decreases in cell number in each layer are equal to the number of cell divisions. Thus, the basal cell density, the number of basal cell divisions, and the frequency of oblique divisions are all increased in thick epidermis compared with thin epidermis. This result indicates that the upward stream is faster in thick epidermis (Fig 7B). Accordingly, more cells are supplied to the thick epidermis from the basal layer to the suprabasal layer, where the cells shift to a differentiated state. Because of the efficient supply of the differentiated suprabasal cells, the oblique division can be considered to play a key role in maintaining epidermal thickness.

The orientation of cell division during development of the epidermis is thought to be regulated by the intracellular localization of several molecules, such as Gα_i_, mInsc (mammalian homologue of Inscuteable), LGN (Leu-Gly-Asn repeat-enriched protein), NuMA (nuclear mitotic apparatus protein), Par-3 (partition-defective 3), and aPKC [12, 14–16]. Additionally, recent study showed that PDK1 (3-phosphoinositede dependent protein kinase-1) plays a crucial role in the recruitment and activation of aPKC and Par-3 at the apical side of dividing basal cells in embryonic mice [17]. Considering the good correlation between epidermal thickness and oblique divisions in adult mice in this study, these molecules may regulate the cell division orientation in adult to maintain the epidermal thickness. In fact, the loss of aPKCλ showed a higher frequency of divisions with an angle of 60–90° and increase in the epidermal thickness [18]. Our observation method would be useful to reveal the molecular mechanisms in the adult epidermis by combining the living transgenic mice that are labeled with or defective in one of the candidate molecules.

In this study, by taking advantage of our imaging technique, we reported the relationship between the morphological features and cell division orientation. Our results provide new insights into maintenance of epidermal thickness. Moreover, we expect that our intravital imaging method will aid in the understanding the mechanisms of epidermal homeostasis.

## Materials and Methods

### Mice

Dr. Toshihiko Fujimori of the National Institute for Basic Biology in Japan provided the R26H2BEGFP mice [26]. R26H2BEGFP hairless mice were obtained by backcrossing the animals with HR-1/Hos mice five times (Hoshino Laboratory Animals, Yoshino, Japan). These mice were maintained by continuous inbreeding to retain a stable genetic background. Male mice aged 8–12 weeks were used for all experiments. All mice used in this study were housed in cages with food and water provided *ad libitum* on a 12-hour light/dark cycle (lights on from 8:00 to 20:00), with controlled temperature and humidity. All mice used for the experiments were euthanized by cervical dislocation. All animal experiments were performed in accordance with the National University Corporation Hokkaido University Regulations on Animal Experimentation and the Guidelines for Proper Conduct of Animal Experiments (Science Council of Japan). The Institutional Animal Care and Use Committee of National University Corporation Hokkaido University approved the protocol (Permit Number: 12–0111). Researchers who wish to use the R26H2BEGFP hairless mice for their studies should contact Dr. Toshihiko Fujimori.

### Histology and immunostaining

The skin harvested from each site was fixed in 4% formaldehyde in phosphate-buffered saline (PBS) at 4°C for 2–3 days. The fixed tissue was embedded in SCEM (Leica Microsystems, Tokyo, Japan) and frozen. The frozen tissues were cut to a 10-μm thickness using a Leica CM3050 S cryostat (Leica Microsystems, Wetzlar, Germany). For the histological analysis, skin sections were stained with hematoxylin and eosin (H&E; Sakura Finetek Japan, Tokyo, Japan) and mounted on coverslips in Multi Mount 480 (Matsunami Glass, Osaka, Japan). Images of the H&E-stained sections were obtained using a bright field microscope (IX70, Olympus, Tokyo, Japan) and a microscope digital camera (DP21, Olympus). For immunostaining, the tissue sections were washed with PBS and 10 mM glycine in PBS and blocked in 3% bovine serum albumin in PBS for 30 min at room temperature. Subsequently, the sections were incubated with an anti-keratin 14 (K14) rabbit polyclonal antibody (Covance, Princeton, NJ) at a dilution of 1:1,000 or an anti-keratin 10 (K10) mouse polyclonal antibody (Thermo Fisher Scientific, Waltham, MA) at a dilution of 1:200 for 1 hour. After washing, an Alexa Fluor 546-conjugated anti-mouse or anti-rabbit IgG antibody (Thermo Fisher Scientific) was used as a secondary antibody at a dilution of 1:400. Hoechst 33342 (Dojindo, Kumamoto, Japan) was used to stain the nuclei. The immunostained sections were photographed using a confocal microscope (A1R, Nikon, Tokyo, Japan) and a CFI Plan Apo VC 60XWI objective lens (Nikon).

### Settings for intravital imaging

Mice were anesthetized via the inhalation of 1.0%–2.0% isoflurane. The wall of a glass-bottomed dish was removed using an ultrasonic cutter to avoid collision with the objective lens. The glass-bottomed dish was fixed on the center of the adapter stage with plastic tape. A ring neodymium magnet (diameter = 12 mm, inner diameter = 8 mm, thickness = 1 mm, weight = 0.46 g; NR108, Nirokuseisakusyo, Kobe, Japan) was fixed as an observation window on the glass-bottomed dish using DS tape A (#04683, Konishi, Osaka, Japan) to observe the dorsal epidermis. Another ring magnet was bonded to one or two sheets of thick paper (thickness = 0.5 mm) with DS tape B (#NW-K15, Nichiban, Tokyo, Japan). Another layer of DS tape A was placed on the other side of the thick paper. This complex was bonded to the gently stretched back skin of the anesthetized mouse on the flipped adapter stage. After a drop of water was placed on the glass-bottomed dish, the back skin was sandwiched between the complex and the ring magnet on the glass-bottomed dish and fixed by the weak magnetic force. The front of the auricle of the anesthetized mice was positioned on the glass-bottomed dish of the flipped adapter stage with a drop of water and stabilized using plastic tape to observe the ear epidermis. The hind paw and tail epidermis were set up similarly to the ear epidermis for observation. The mice were placed on a heating pad to maintain their body temperatures during the observations.

### Intravital imaging

Intravital imaging was performed using an A1RMP+ equipped with gallium arsenide phosphide (GaAsP) non-descanned detectors (Nikon). A UPLFLN4X and an XLPLN25XWMP objective lens (Olympus) were attached to the microscope using a FN-S2N Sliding Nosepiece (Nikon). A two-photon microscope, which can alleviate the influence of scatter and acquire finer images than a confocal microscope, as shown in S6 Fig, was used at an excitation wavelength of 900 nm. An SHG signal at 450 nm from the collagen in the dermis and the EGFP signal were separated and detected using a 458-nm long-pass dichroic mirror and a 560-nm long-pass dichroic mirror. Magnified three-dimensional images were acquired over 50 optical sections with a view field of 1,024×1,024 pixels (0.224 μm/pixel) in 1-μm z-steps at 2 sec/frame every 3 min.

### Software for data analysis

NIS-Elements software (Nikon) was used for data analysis. All time-lapse images were processed using Imaris software (Oxford Instruments, Oxfordshire, UK) to correct the slight drift that occurred during the observations. Movies were created using Imaris and NIS-Elements software. The Steel-Dwass test was performed with the statistical programming language R.

### Measurement of the epidermal thickness

Epidermal thickness (without the cornified layer) and the thickness of the cornified layer shown in S1 Fig were determined from the images of H&E-stained sections of R26H2BEGFP hairless mice and the littermates. The statistical significance of the difference between the R26H2BEGFP hairless mice and the littermates was assessed using the Steel-Dwass test, as shown in S1 and S2 Tables. The epidermal thickness (without the cornified layer) and the thickness of the cornified layer shown in Fig 3 were determined from three-dimensional images obtained by intravital skin imaging of R26H2BEGFP hairless mice. The statistical significance of the differences between the body regions was assessed using the Steel-Dwass test, as shown in S3 and S4 Tables.

### Measurement of the cell density

The basal cell numbers shown in Fig 3 were scored from three-dimensional images that were cropped to exclude the hair follicles and the cell density was calculated. The statistical significance of the differences between body regions was assessed using the Steel-Dwass test, as shown in S5 Table.

### Measurement of the cell division orientation

Specific criteria for each body site were determined to measure the lateral cell division orientation, as shown in S5 Fig. The angle between the reference line and the lateral component of the line that connected the pair of nuclei of the daughter cells was measured in the range from 0–90°. The cell division orientation relative to the basement membrane was measured as shown in Fig 6A. Specifically, sequential images were rotated around the *z*-axis by the angle between the *x*-axis and the lateral component of the cell division orientation. Subsequently, the angle between the lines along the basement membrane was defined by the SHG signals, and the line that connects the pair of nuclei was measured from the orthogonally viewed image.

## Supporting Information

S1 Fig Establishment of R26H2BEGFP hairless mice for intravital epidermal imaging. (**A**) H&E-stained sections of the dorsal, ear, hind paw, and tail skin. The arrows indicate the scale region and the arrowheads show the interscale region of the tail epidermis. The skin of the R26H2BEGFP hairless mice showed no apparent abnormalities compared with the WT mice. (**B**, **C**) Thickness of the epidermis without the cornified layer (**B**) and the thickness of the cornified layer (**C**) were measured in H&E-stained sections for each epidermal tissue. These data were obtained from at least 18 points across 4–5 mice per group and compared using the Steel-Dwass test. The error bars represent the standard deviations *P < 0.05; n.s., not significant. See also S1 and S2 Tables. (**D**) Immunofluorescently (K14 or K10 and Hoechst 33342) stained sections of each body site in the R26H2BEGFP hairless mice. The localization of K14 and K10 appeared to be normal. Scale bar = 50 μm.
(PDF)

S2 Fig Detailed procedure of our method for holding dorsal skin. (**A**) The wall of a glass-bottomed dish was cut to avoid collision with the objective lens. A ring magnet was attached on the center of the cut glass-bottomed dish using ring-shaped DS tape A. (**B**) The cut glass-bottomed dish was placed on the center of the adapter stage and fixed with plastic tape, and the adapter stage was inverted. (**C**) The other ring magnet, DS tape B, thick paper, and DS tape A were superimposed to form a complex. (**D**) The anaesthetized mouse was placed on the inverted adapter stage with the cut glass-bottomed dish. The dorsal skin of the mouse was slightly strained and sandwiched between a pair of ring magnets.
(PDF)

S3 Fig Three-dimensional imaging of the dorsal, ear, hind paw, and tail epidermis in living R26H2BEGFP hairless mice. (**A**, **C**, **D**, **F**, **G**, **I**) Low-magnification images of the dorsum (**A**), ear (**C**, **D**), hind paw (**F**, **G**), and tail (**I**) were obtained using a confocal microscope. Scale bar = 1 mm. (**B**, **E**, **H**, **J**) Optically sectioned images of the yellow square regions in **A**, **D**, **G** and **I**, respectively, using a two-photon microscope. The depth from the surface of the skin is shown in the upper left of the images. The white asterisks indicate the hair follicles. Scale bar = 50 μm.
(PDF)

S4 Fig Changes in the chromatin structures via intravital four-dimensional imaging. (**A**) Time-lapse image of the dorsal epidermis at 0 min. The red rectangles show the cells that divided during the 4-hour imaging session. Scale bar = 50 μm. (**B**) Sequential images of each of the four dividing cells during the time-lapse imaging of the areas indicated by the red rectangles in **A**. Scale bar = 5 μm. A movie of the fluorescent images is available as S1 Movie.
(PDF)

S5 Fig Analysis of the lateral component of the cell division orientation. (**A**, **C**, **E**, **G**) Schematic of the measurement of the lateral component of the cell division orientation in each epidermis. Each blue line shows the reference line that was defined based on specific criteria. In the dorsum (**A**) and tail (**G**), the reference line was defined as the hair follicle orientation, which was approximately equal to the body axis. In the ear (**C**), the reference line was also defined as the hair follicle orientation, which approximately indicates a radial orientation relative to the ear edge. The reference line of the hind paw was determined as the line from the center of the heel to the middle toe (**E**). (**B**, **D**, **F**, **H**) The lateral angle distribution of the cell divisions in the dorsal (**B**), ear (**D**), hind paw (**F**), and tail (interscale and scale) (**H**) epidermis.
(PDF)

S6 Fig Comparison of confocal and two-photon microscopy for intravital skin imaging using a R26H2BEGFP hairless mouse. (**A**, **B**) Confocal images of the *x*-*y* and *x*-*z* planes of the dorsal (**A**) and hind paw (**B**) epidermis, with the pinhole set to 1 airy unit. (**C**, **D**) Two-photon images of the *x*-*y* and *x*-*z* planes of the dorsal (**C**) and hind paw (**D**) epidermis. Scale bar = 10 μm.
(PDF)

S1 Movie Cropped movie of the observation area of the dorsal epidermis (left panel) and magnified movies of four cell divisions (right panels) in the areas indicated by red rectangles in the left panel. The movie was recorded at 3 minutes per frame and is playing at 70 ms/frame (1,500-fold enhanced speed). See S4 Fig.
(MOV)

S2 Movie Cropped movie of a cell division in the *x*-*y* plane at a depth of 24 μm from the skin surface (left panel) and three-dimensional representation (right panel) of dorsal epidermis. The red arrows indicate a dividing cell. The movie was recorded at 3 minutes per frame and is playing at 70 ms/frame (1,500-fold enhanced speed). See Fig 4.
(MOV)

S3 Movie Cropped movie of an oblique division in the *x*-*y* plane at three different depths from the skin surface (left panel) and three-dimensional representation (right panel) of the hind paw epidermis. The red arrows indicate a dividing cell. The movie was recorded at 3 minutes per frame and is playing at 70 ms/frame (1,500-fold enhanced speed). See Fig 5A and 5B.
(MOV)

S4 Movie Cropped movie of a parallel division in the *x*-*y* plane at a depth of 32 μm from the skin surface (left panel) and three-dimensional representation (right panel) of the hind paw epidermis. The red arrows indicate a dividing cell. The movie was recorded at 3 minutes per frame and is playing at 70 ms/frame (1,500-fold enhanced speed). See Fig 5C and 5D.
(MOV)

S1 Table Statistical significance of differences between the thickness of the epidermis without the cornified layer between the R26H2BEGFP hairless mice and littermates using the Steel-Dwass test (See S1B Fig). (PDF)

S2 Table Statistical significance of the differences in the thickness of the cornified layer between the R26H2BEGFP hairless mice and littermates (See S1C Fig). (PDF)

S3 Table Statistical significance of the differences in the thickness of the epidermis without the cornified layer between body regions using the Steel-Dwass test (See Fig 3I). (PDF)

S4 Table Statistical significance of the differences in the thickness of the cornified layer between body regions using the Steel-Dwass test (See Fig 3J). (PDF)

S5 Table Statistical significance of the differences in the basal cell density between body regions using the Steel-Dwass test (See Fig 3K). (PDF)
