# Supplementary figures and images for: Three-Dimensional Analysis of Cell Division Orientation in Epidermal Basal Layer Using Intravital Two-Photon Microscopy
Source: PLoS One. 2016 Sep 22;11(9):e0163199. doi: 10.1371/journal.pone.0163199 (PMC5033459; doi:10.1371/journal.pone.0163199)

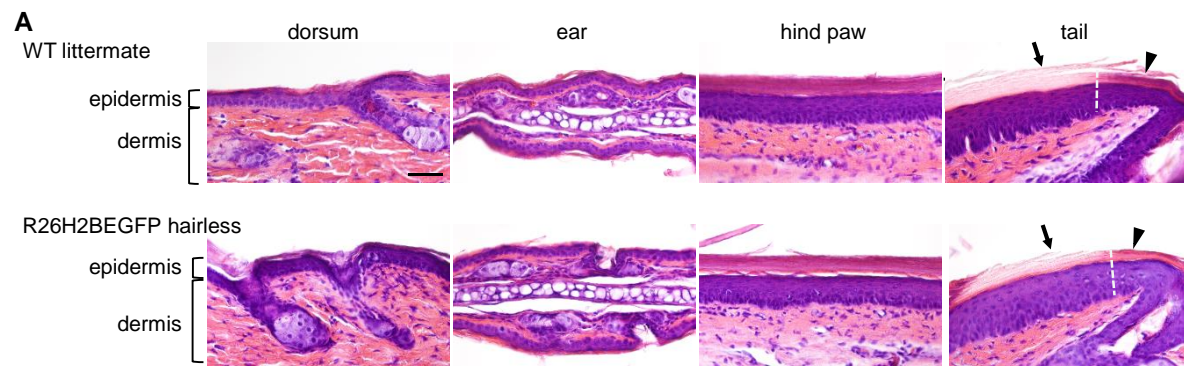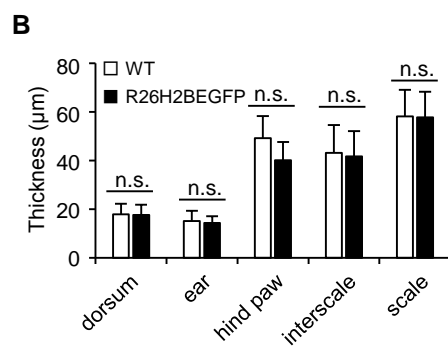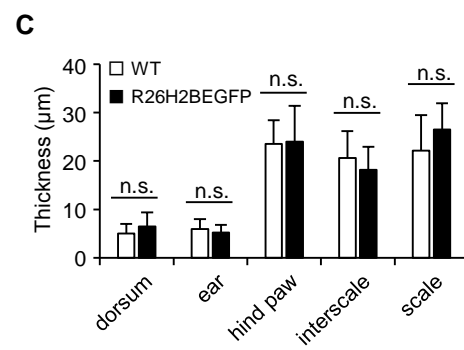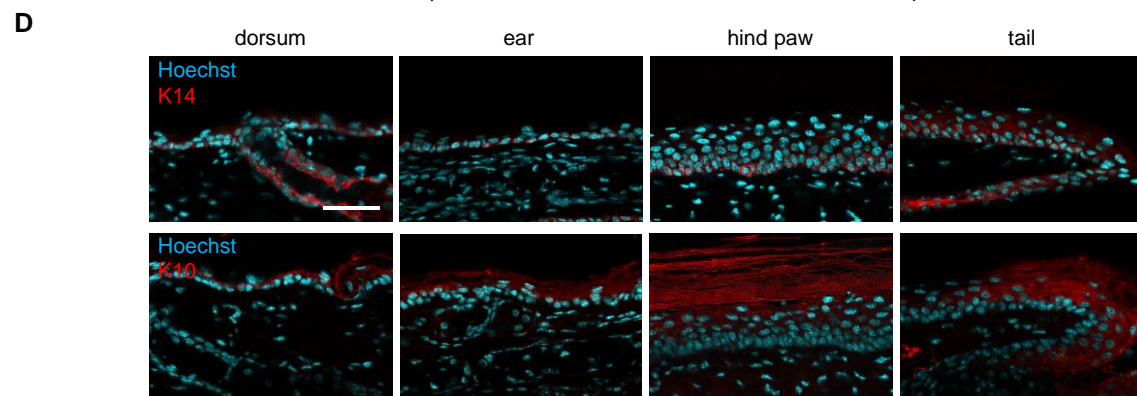

Supplement: S1 Fig — (A) H&E-stained sections of the dorsal, ear, hind paw, and tail skin. The arrows indicate the scale region and the arrowheads show the interscale region of the tail epidermis. The skin of the R26H2BEGFP hairless mice showed no apparent abnormalities compared with the WT mice. (B, C) Thickness of the epidermis without the cornified layer (B) and the thickness of the cornified layer (C) were measured in H&E-stained sections for each epidermal tissue. These data were obtained from at least 18 points across 4–5 mice per group and compared using the Steel-Dwass test. The error bars represent the standard deviations *P < 0.05; n.s., not significant. See also S1 and S2 Tables. (D) Immunofluorescently (K14 or K10 and Hoechst 33342) stained sections of each body site in the R26H2BEGFP hairless mice. The localization of K14 and K10 appeared to be normal. Scale bar = 50 μm. (PDF) [file pone.0163199.s001.pdf]

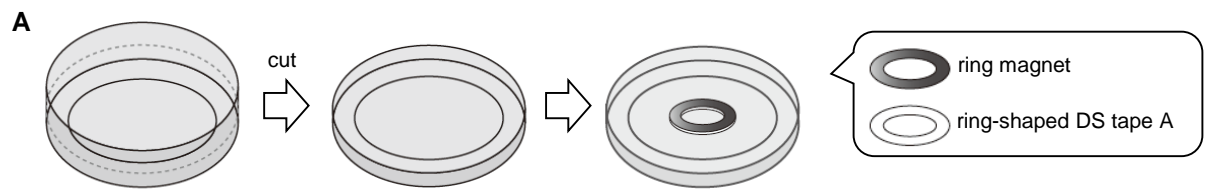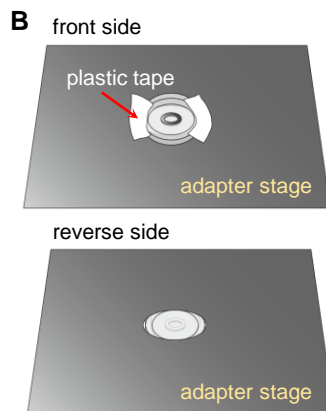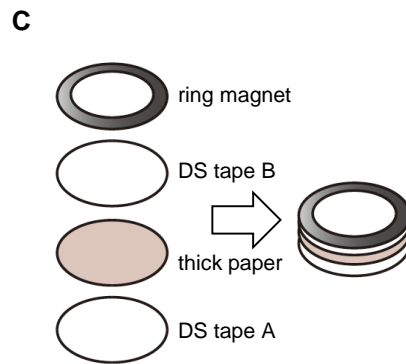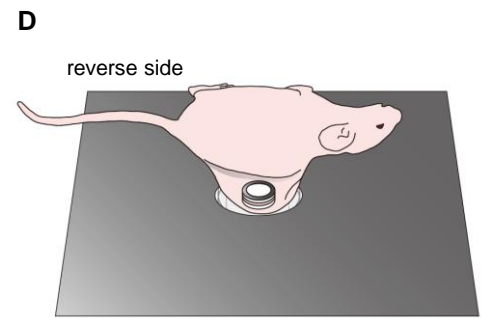

Supplement: S2 Fig — (A) The wall of a glass-bottomed dish was cut to avoid collision with the objective lens. A ring magnet was attached on the center of the cut glass-bottomed dish using ring-shaped DS tape A. (B) The cut glass-bottomed dish was placed on the center of the adapter stage and fixed with plastic tape, and the adapter stage was inverted. (C) The other ring magnet, DS tape B, thick paper, and DS tape A were superimposed to form a complex. (D) The anaesthetized mouse was placed on the inverted adapter stage with the cut glass-bottomed dish. The dorsal skin of the mouse was slightly strained and sandwiched between a pair of ring magnets. (PDF) [file pone.0163199.s002.pdf]

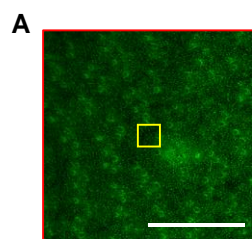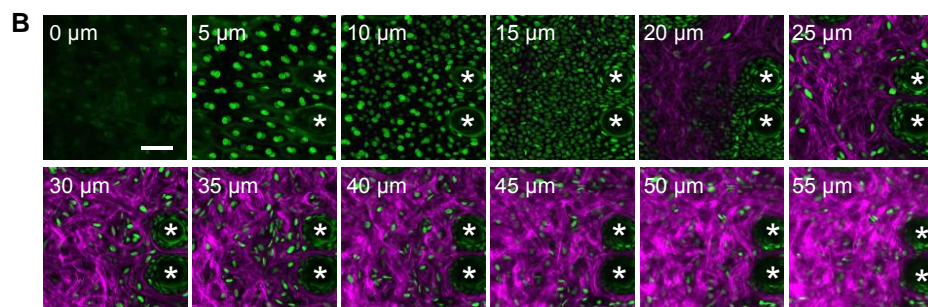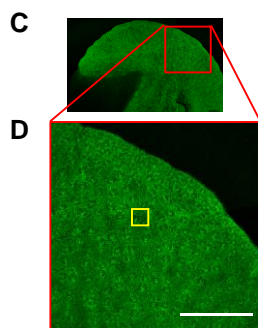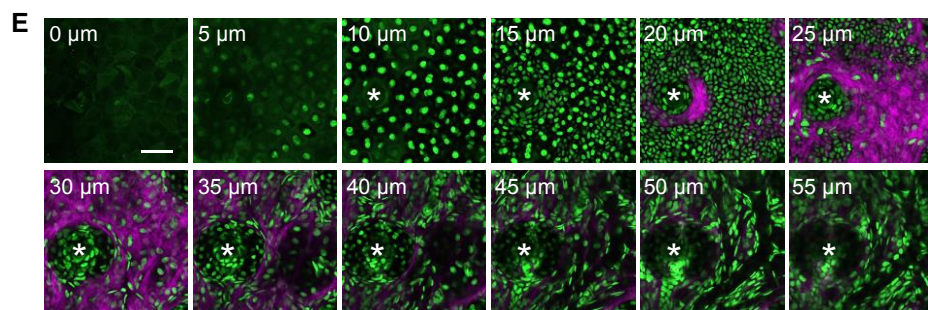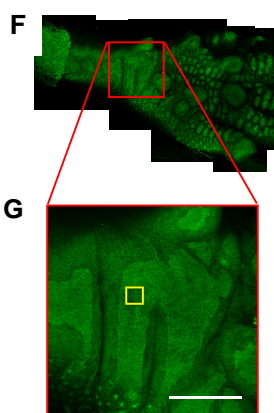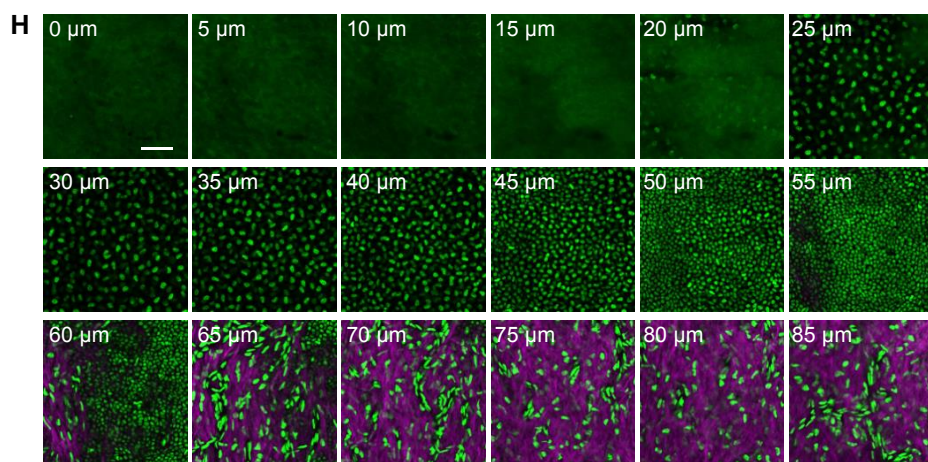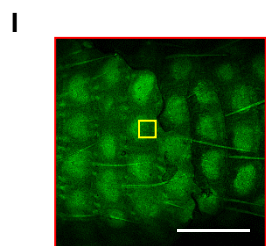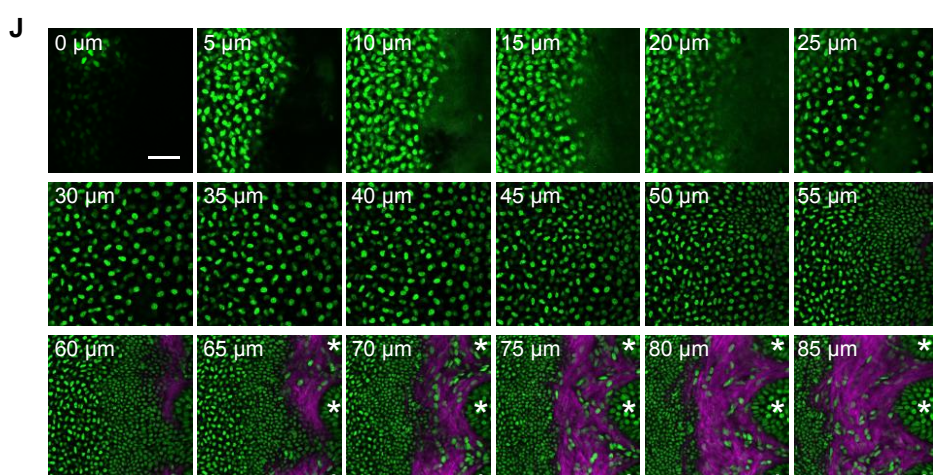

Supplement: S3 Fig — (A, C, D, F, G, I) Low-magnification images of the dorsum (A), ear (C, D), hind paw (F, G), and tail (I) were obtained using a confocal microscope. Scale bar = 1 mm. (B, E, H, J) Optically sectioned images of the yellow square regions in A, D, G and I, respectively, using a two-photon microscope. The depth from the surface of the skin is shown in the upper left of the images. The white asterisks indicate the hair follicles. Scale bar = 50 μm. (PDF) [file pone.0163199.s003.pdf]

**A**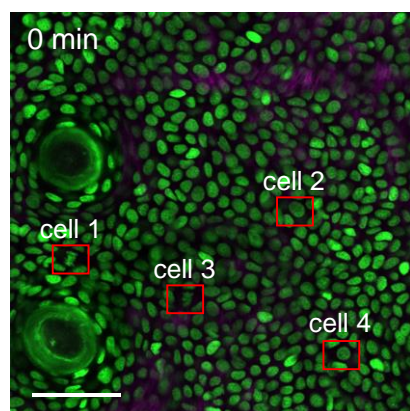**B**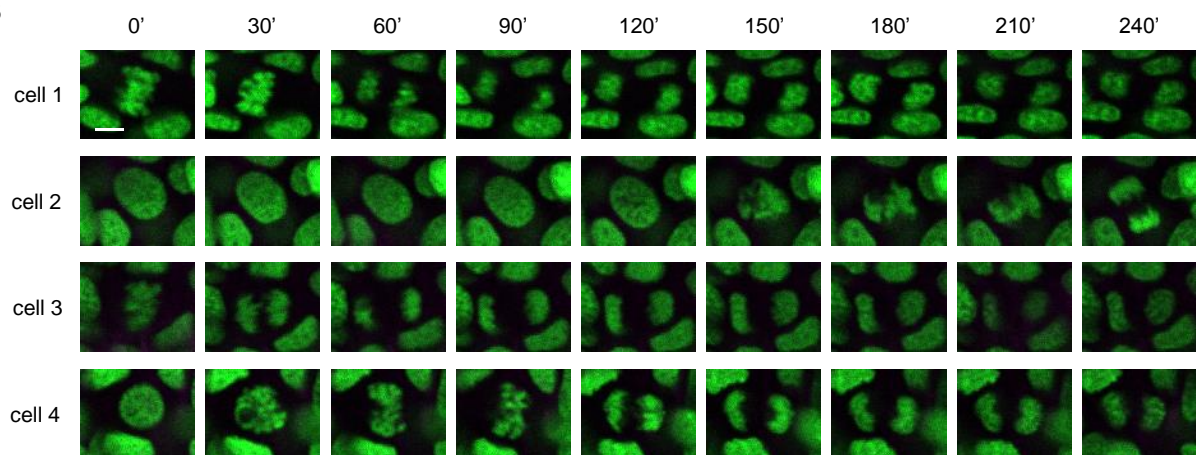

Supplement: S4 Fig — (A) Time-lapse image of the dorsal epidermis at 0 min. The red rectangles show the cells that divided during the 4-hour imaging session. Scale bar = 50 μm. (B) Sequential images of each of the four dividing cells during the time-lapse imaging of the areas indicated by the red rectangles in A. Scale bar = 5 μm. A movie of the fluorescent images is available as S1 Movie. (PDF) [file pone.0163199.s004.pdf]

**A** dorsum

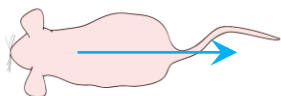

**C** ear

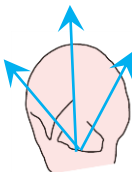

**E** hind paw

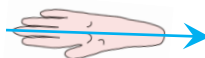

**G** tail

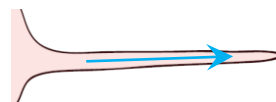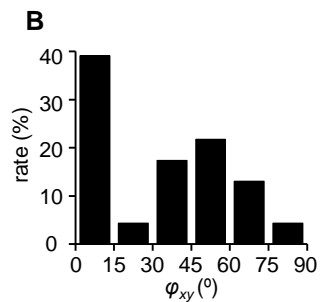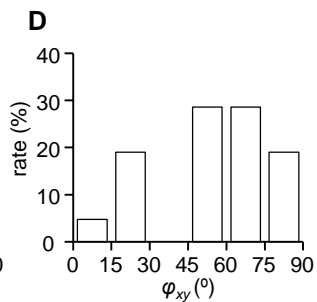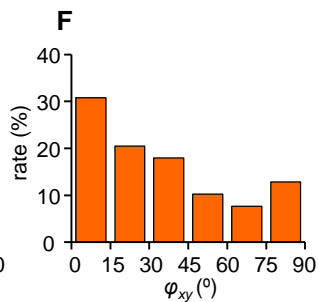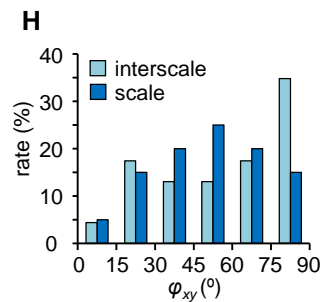

Supplement: S5 Fig — (A, C, E, G) Schematic of the measurement of the lateral component of the cell division orientation in each epidermis. Each blue line shows the reference line that was defined based on specific criteria. In the dorsum (A) and tail (G), the reference line was defined as the hair follicle orientation, which was approximately equal to the body axis. In the ear (C), the reference line was also defined as the hair follicle orientation, which approximately indicates a radial orientation relative to the ear edge. The reference line of the hind paw was determined as the line from the center of the heel to the middle toe (E). (B, D, F, H) The lateral angle distribution of the cell divisions in the dorsal (B), ear (D), hind paw (F), and tail (interscale and scale) (H) epidermis. (PDF) [file pone.0163199.s005.pdf]

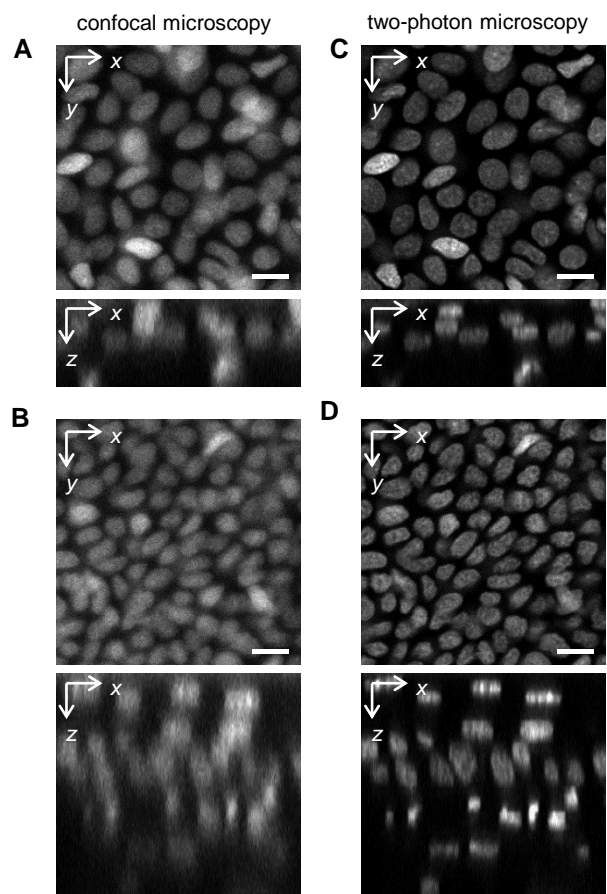

Supplement: S6 Fig — (A, B) Confocal images of the x-y and x-z planes of the dorsal (A) and hind paw (B) epidermis, with the pinhole set to 1 airy unit. (C, D) Two-photon images of the x-y and x-z planes of the dorsal (C) and hind paw (D) epidermis. Scale bar = 10 μm. (PDF) [file pone.0163199.s006.pdf]
