# Supplementary material for: Three-Dimensional Analysis of Cell Division Orientation in Epidermal Basal Layer Using Intravital Two-Photon Microscopy
Source: PLoS One. 2016 Sep 22;11(9):e0163199. doi: 10.1371/journal.pone.0163199 (PMC5033459; doi:10.1371/journal.pone.0163199)
Supplement: S1 Table — (PDF) [file pone.0163199.s011.pdf]

**S1 Table. Statistical significance of differences between the thickness of the epidermis without the cornified layer between the R26H2BEGFP hairless mice and littermates using the Steel-Dwass test (See S1B Fig).**

| | dorsum (L) | dorsum (G) | ear (L) | ear (G) | hind paw (L) | hind paw (G) | interscale (L) | interscale (G) | scale (L) | scale (G) |
|---|---|---|---|---|---|---|---|---|---|---|
| dorsum (L) | - | n.s. | n.s. | n.s. | ** | ** | ** | ** | ** | ** |
| dorsum (G) | - | - | n.s. | n.s. | ** | ** | ** | ** | ** | ** |
| ear (L) | - | - | - | n.s. | ** | ** | ** | ** | ** | ** |
| ear (G) | - | - | - | - | ** | ** | ** | ** | ** | ** |
| hind paw (L) | - | - | - | - | - | n.s. | n.s. | n.s. | ** | ** |
| hind paw (G) | - | - | - | - | - | - | n.s. | n.s. | n.s. | n.s. |
| interscale (L) | - | - | - | - | - | - | - | n.s. | ** | ** |
| interscale (G) | - | - | - | - | - | - | - | - | ** | ** |
| scale (L) | - | - | - | - | - | - | - | - | - | n.s. |
| scale (G) | - | - | - | - | - | - | - | - | - | - |

(G) R26H2BEGFP hairless mice (L) littermates ** $P < 0.01$ * $P < 0.05$ n.s. $P \geq 0.05$
