# Supplementary material for: Three-Dimensional Analysis of Cell Division Orientation in Epidermal Basal Layer Using Intravital Two-Photon Microscopy
Source: PLoS One. 2016 Sep 22;11(9):e0163199. doi: 10.1371/journal.pone.0163199 (PMC5033459; doi:10.1371/journal.pone.0163199)
Supplement: S2 Table — (PDF) [file pone.0163199.s012.pdf]

S2 Table. Statistical significance of the differences in the thickness of the cornified layer between the R26H2BEGFP hairless mice and littermates (See S1C Fig).

	dorsum (L)	dorsum (G)	ear (L)	ear (G)	hind paw (L)	hind paw (G)	interscale (L)	interscale (G)	scale (L)	scale (G)
dorsum (L)	-	n.s.	n.s.	n.s.	**	**	**	**	**	**
dorsum (G)	-	-	n.s.	n.s.	**	**	**	**	**	**
ear (L)	-	-	-	n.s.	**	**	**	**	**	**
ear (G)	-	-	-	-	**	**	**	**	**	**
hind paw (L)	-	-	-	-	-	n.s.	n.s.	n.s.	n.s.	n.s.
hind paw (G)	-	-	-	-	-	-	*	n.s.	n.s.	n.s.
interscale (L)	-	-	-	-	-	-	-	n.s.	**	n.s.
interscale (G)	-	-	-	-	-	-	-	-	*	n.s.
scale (L)	-	-	-	-	-	-	-	-	-	n.s.
scale (G)	-	-	-	-	-	-	-	-	-	-

(G) R26H2BEGFP hairless mice (L) littermates ** $P < 0.01$ * $P < 0.05$ n.s. $P \geq 0.05$
