# Supplementary material for: Three-Dimensional Analysis of Cell Division Orientation in Epidermal Basal Layer Using Intravital Two-Photon Microscopy
Source: PLoS One. 2016 Sep 22;11(9):e0163199. doi: 10.1371/journal.pone.0163199 (PMC5033459; doi:10.1371/journal.pone.0163199)
Supplement: S5 Table — (PDF) [file pone.0163199.s015.pdf]

S5 Table. Statistical significance of the differences in the basal cell density between body regions using the Steel-Dwass test (See Fig 3K).

	dorsum	ear	hind paw	interscale	scale
dorsum	-	n.s.	*	n.s.	**
ear	-	-	*	n.s.	*
hind paw	-	-	-	*	n.s.
interscale	-	-	-	-	**
scale	-	-	-	-	-

** $P < 0.01$ * $P < 0.05$ n.s. $P \geq 0.05$
